# Supplementary material for: Bayesian-calibrated global sensitivity analysis for mathematical models using generative AI
Source: PLoS Comput Biol. 2026 Mar 16;22(3):e1013312. doi: 10.1371/journal.pcbi.1013312 (PMC13004599; doi:10.1371/journal.pcbi.1013312)
Supplement: S1 Appendix — Supplementary results comparing the true input distribution with the generative model–learned distribution, together with detailed GSA results and bootstrap confidence intervals. (PDF) [file pcbi.1013312.s001.pdf]

S1 Appendix. Linear Function. In the main manuscript, the distribution parameters used for the input variable $\boldsymbol{X}$ are given by

$$\boldsymbol{\mu} = \boldsymbol{0}, \quad \Sigma = \begin{bmatrix} 0.04 & 0.06 & 0.10 \\ 0.06 & 0.36 & 0.30 \\ 0.10 & 0.30 & 1.00 \end{bmatrix}.$$

To illustrate how Sobol's two-matrix estimator fails to preserve input correlations, we compare pairwise scatter plots of samples drawn from the true input distribution $\mathcal{N}(\boldsymbol{\mu}, \boldsymbol{\Sigma})$ with those generated by Sobol's method. Fig A presents the pairwise scatter plots for both cases, each based on 2,000 samples of $\boldsymbol{X}$. Since the assumption of input independence is violated, both Sobol's method and the PCE method are expected to perform poorly in estimating the total order sensitivity indices. A detailed comparison based on 2,000 bootstrap replicates is presented in Fig B.

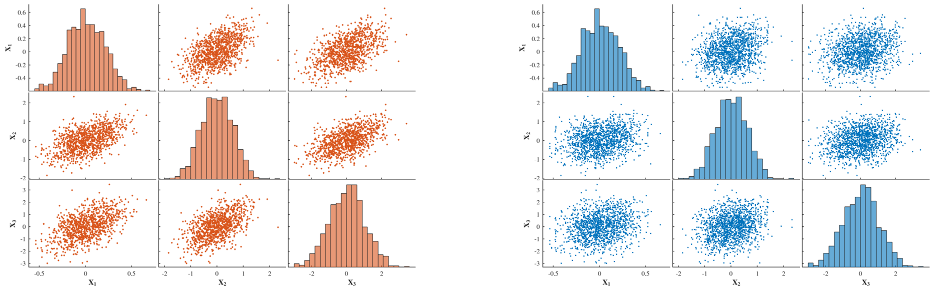

A. True input distribution **B.** Sobol's two-matrix estimator

Fig. A. Comparison. Input sample structures used for sensitivity analysis: (a) samples drawn from the true correlated input distribution, and (b) conditional samples generated by Sobol's two-matrix estimator via column swapping between independent sample matrices.

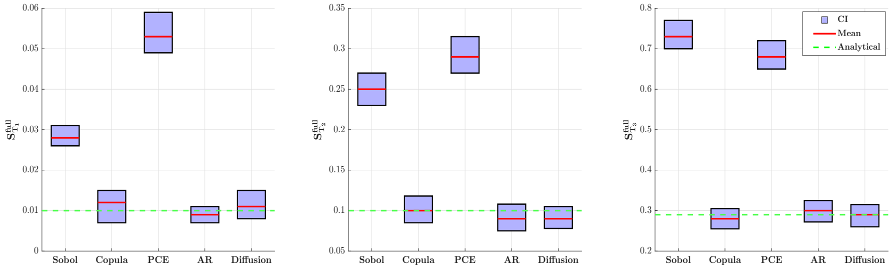

Fig. B. Results. Comparison of the full total order sensitivity indices for the linear function, computed using a bootstrap with 2,000 replicates.
