# Supplementary material for: Bayesian-calibrated global sensitivity analysis for mathematical models using generative AI
Source: PLoS Comput Biol. 2026 Mar 16;22(3):e1013312. doi: 10.1371/journal.pcbi.1013312 (PMC13004599; doi:10.1371/journal.pcbi.1013312)
Supplement: S2 Appendix — Supplementary results comparing the true input and conditional distributions with the learned generative distributions, including effective sample size diagnostics and detailed GSA results with bootstrap confidence intervals. (PDF) [file pcbi.1013312.s002.pdf]

S2 Appendix. Ishigami Function. This supplement provides additional results from the benchmark study of the Ishigami function. The input vector $\boldsymbol{X}$ exhibits a dependence structure specified by a C-vine copula, which is not expected to be well approximated by Gaussian conditional densities. Fig A presents pairwise scatter plots of samples drawn from the true input distribution, together with samples generated by different surrogate models. It is evident that both the Gaussian copula and the autoregressive model with Gaussian conditionals fail to capture the heavy-tailed dependence structure present in the true distribution, whereas the diffusion model provides a much closer approximation.

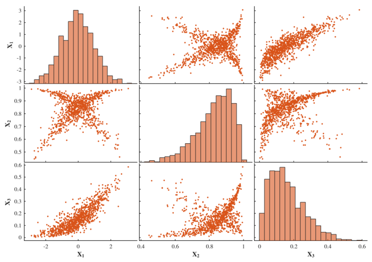

A. True input distribution

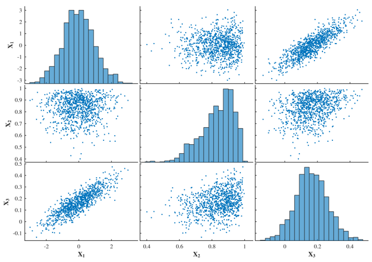

B. Gaussian copula

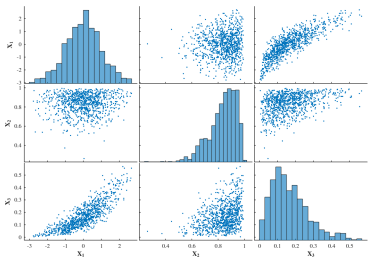

C. Autoregressive model

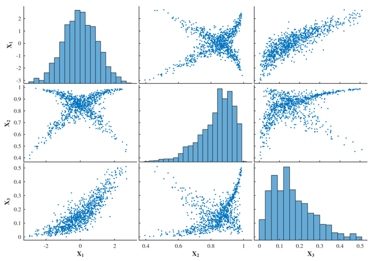

D. Diffusion model

Fig. A. Comparison. True input distribution for the Ishigami function versus distributions learned using different GSA methods.

As discussed in the main text, the estimation of sensitivity indices requires surrogate models to generate conditional samples. We therefore compare the ability of the autoregressive and diffusion models to accurately reproduce conditional distributions. Fig B shows the estimated conditional densities alongside the true conditional densities, obtained by fixing $X_3 = 0.4$. The autoregressive model, which assumes Gaussian conditional densities, captures unimodal conditional distributions reasonably well, but fails to reproduce the bimodal structure of $p(X_2 \mid X_3 = 0.4)$. In contrast, the diffusion model combined with the Repaint algorithm successfully recovers the overall shape of the conditional densities, although a small bias remains due to the heuristic nature of the Repaint procedure. The resulting GSA results, including detailed bootstrap confidence intervals, are presented in Fig C.

In addition to the sensitivity analysis results, we further investigate the choice of neural network architecture and the sample size required to accurately recover the input distribution. Table A and Table B present the learned input densities obtained using different neural network architectures and training sample sizes, with the Kullback–Leibler (KL) divergence used as an error metric to quantify the discrepancy between the learned and true input distributions. The KL divergence is estimated using

kernel density estimation based on 5,000 samples. Highlighted values indicate the minimal effective sample size, defined as the point at which subsequent increases in the training set size result in an error reduction of less than 0.01.

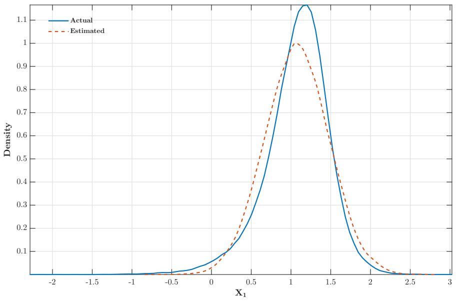

A. Autoregressive model

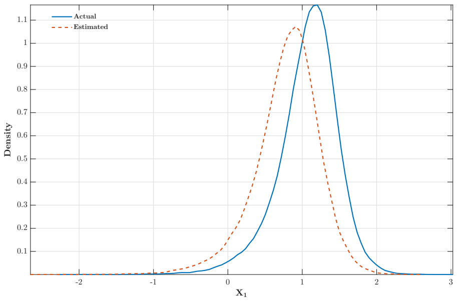

B. Diffusion model

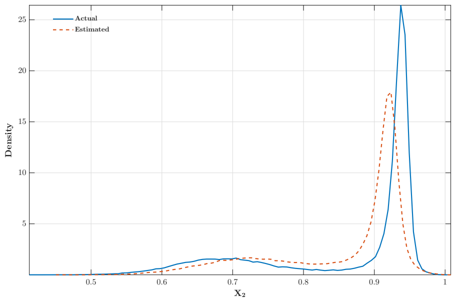

C. Autoregressive model

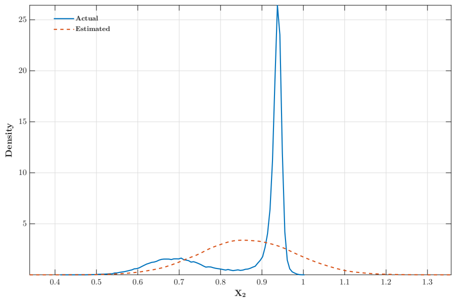

D. Diffusion model

Fig. B. Comparison. True conditional density of the Ishigami function given $X_3 = 0.4$, compared with estimates obtained using different generative models.

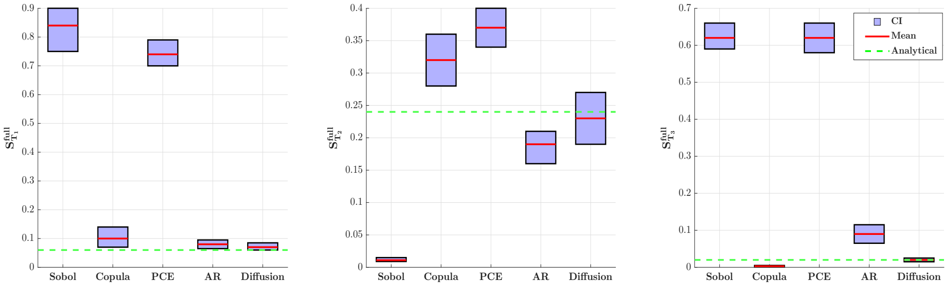

Fig. C. Results. Comparison of the full total order sensitivity indices for the Ishigami function, computed using a bootstrap with 2,000 replicates.

Table A. Performance comparison of recovering the true input distribution using varying numbers of training samples and neural network architectures (layers × neurons) in autoregressive model. Errors are measured by the KL divergence.

Samples	2×8	2×16	2×32	2×64	2×256
2000	0.150	0.160	0.185	0.210	0.280
4000	**0.138**	0.125	0.110	0.135	0.180
6000	0.135	**0.112**	**0.068**	0.095	0.130
8000	0.133	0.108	0.065	0.070	0.100
10000	0.132	0.105	0.063	0.055	0.075
20000	0.131	0.103	0.061	**0.040**	0.045
50000	0.130	0.102	0.060	0.035	**0.025**

Table B. Performance comparison of recovering the true input distribution using varying numbers of training samples and neural network architectures (layers × neurons) in diffusion model. Errors are measured by the KL divergence.

Samples	2×8	2×16	2×32	2×64	2×256
2000	0.185	0.190	0.220	0.280	0.350
4000	**0.172**	**0.145**	0.160	0.190	0.260
6000	0.170	0.142	**0.082**	0.130	0.190
8000	0.169	0.140	0.080	0.095	0.150
10000	0.168	0.139	0.078	**0.072**	0.110
20000	0.168	0.138	0.076	0.065	0.060
50000	0.167	0.137	0.075	0.060	**0.038**
