# Supplementary material for: Bayesian-calibrated global sensitivity analysis for mathematical models using generative AI
Source: PLoS Comput Biol. 2026 Mar 16;22(3):e1013312. doi: 10.1371/journal.pcbi.1013312 (PMC13004599; doi:10.1371/journal.pcbi.1013312)
Supplement: S3 Appendix — Supplementary results assessing the efficiency of generative model based GSA, together with detailed bootstrap GSA estimates. This appendix also includes conditional density estimation results for autoregressive and diffusion models. (PDF) [file pcbi.1013312.s003.pdf]

**S3 Appendix. Sobol Function.** This supplement provides additional results for the benchmark study of the Sobol $g$-function. In Fig A, we present the GSA results for a 200 dimensional Sobol function, together with detailed sensitivity estimates and their associated bootstrap confidence intervals. We further provide a convergence comparison of two Monte Carlo based approaches, namely Algorithms 1 and 2, conducted under the same computational budget. The results show that Algorithm 1 converges more slowly than Algorithm 2, but produces narrower confidence intervals, indicating lower estimation uncertainty. In contrast, Algorithm 2 exhibits larger uncertainty due to the heuristic nature of the Repaint algorithm and the use of fixed and limited inner and outer sample sizes, $N_I$ and $N_O$.

For the Sobol function example, the input variables are independently and uniformly distributed over the unit hypercube. Consequently, for any pair of inputs $X_i$ and $X_j$ with $i \neq j$, the conditional distribution is $p(X_i \mid X_j) = \mathbf{1}_{X_i \in [0,1]}$, where $\mathbf{1}$ denotes the indicator function. This property enables us to assess the accuracy of the conditional generative models by evaluating the average conditional density error across all variable pairs $(i, j)$ with $i, j \in \{1, 2, \ldots, d\}$ and $i \neq j$. Specifically, we evaluate $p(X_i \mid X_j)$ on a uniform grid over $[-0.1, 1.1] \times [-0.1, 1.1]$ to capture both the support region $[0, 1] \times [0, 1]$ and regions outside the support where the conditional density should be zero. The results are summarized in Fig B. From the figure, we observe that the autoregressive model achieves superior performance, as the target distribution can be exactly represented. In contrast, for the diffusion based model, the Repaint algorithm exhibits noticeable errors near the boundaries of the interval, indicating bias in conditional sample generation process.

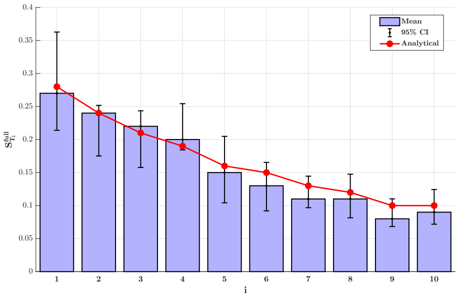


**A.** Sobol's method

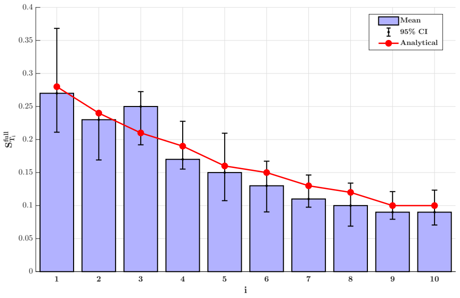


**B.** Gaussian copula

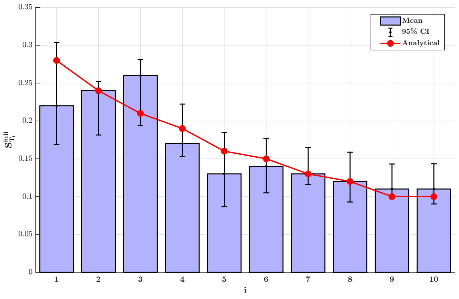


**C.** PCE

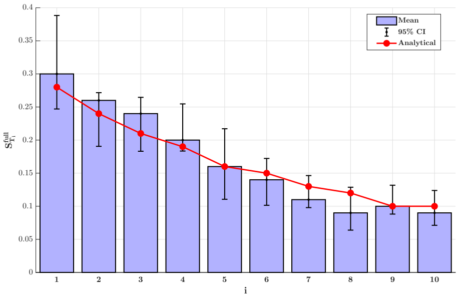


**D.** Autoregressive model

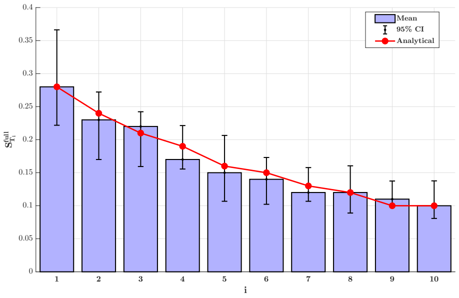


**E.** Diffusion model

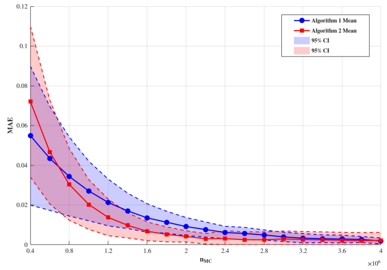


**F.** Monte Carlo convergence

**Fig. A. Results**. GSA for the Sobol $g$-function. Sensitivity indices are estimated using a bootstrap procedure with 2,000 replicates. The convergence plot compares Monte Carlo efficiency across methods under an equal computational budget.

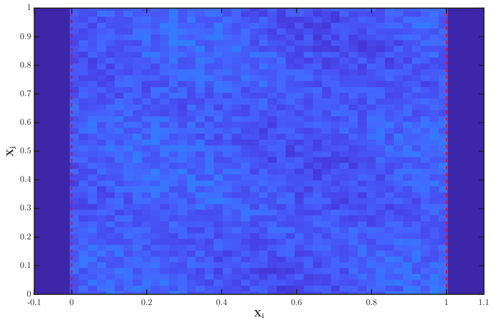

**A.** Autoregressive model

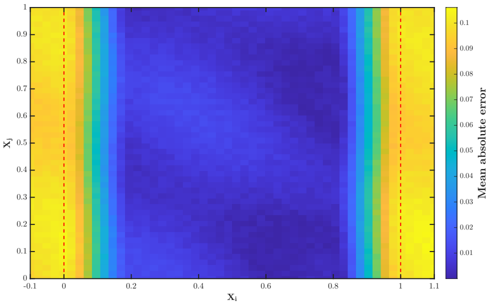


**B.** Diffusion model

**Fig. B. Comparison**. Averaged conditional density estimation errors for the Sobol $g$-function obtained using different generative models.
