# Supplementary material for: Bayesian-calibrated global sensitivity analysis for mathematical models using generative AI
Source: PLoS Comput Biol. 2026 Mar 16;22(3):e1013312. doi: 10.1371/journal.pcbi.1013312 (PMC13004599; doi:10.1371/journal.pcbi.1013312)
Supplement: S4 Appendix — Supplementary results providing detailed parameter definitions and Bayesian calibration results for the SIR model. (PDF) [file pcbi.1013312.s004.pdf]

S4 Appendix. Epidemiology Model. In this supplement, we present the model calibration results in the table below. Based on the calibration results, the posterior predictions for daily new cases are provided in Fig A.

Table A. Calibration results for the SIR model.

Parameter	Description	MLE	95% Credible Interval
β	Transmission rate	1.63×10^{-7}	$(1.32 \times 10^{-7}, 1.67 \times 10^{-7})$
ρ	Case detection rate	1.22×10^{-3}	$(8.43 \times 10^{-4}, 1.77 \times 10^{-3})$
γ	Mean recovery rate	0.0697	$(9.21 \times 10^{-3}, 0.138)$
I_0	Initial infectious population	1.13	(1.53, 41.5)
ϕ	Data precision ($1/\phi$ is variance)	3.40×10^{-7}	$(2.61 \times 10^{-7}, 4.56 \times 10^{-7})$

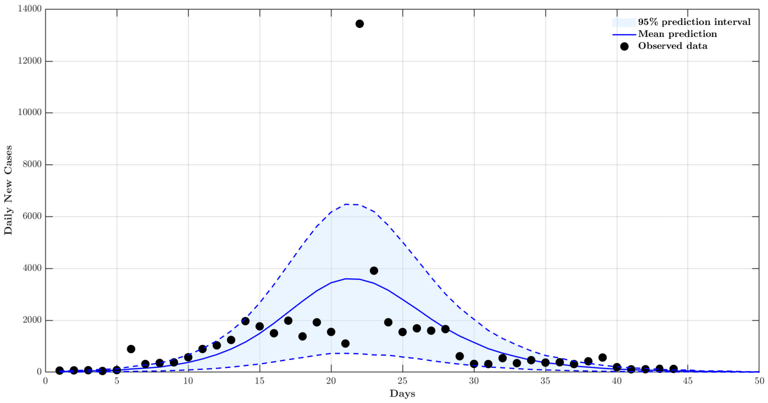

Fig. A. Result. Posterior predictive solutions for daily new detected case number

Using the posterior samples, autoregressive models with different variable permutations are trained. The loss curves for these models are shown in Fig B. Based on the test error, we confirm that the performance of the autoregressive model depends on the order used to decompose the joint distribution. In particular, the higher convergent loss observed for permutation π_3 can be attributed to the heavy-tailed marginal distribution of I_0.

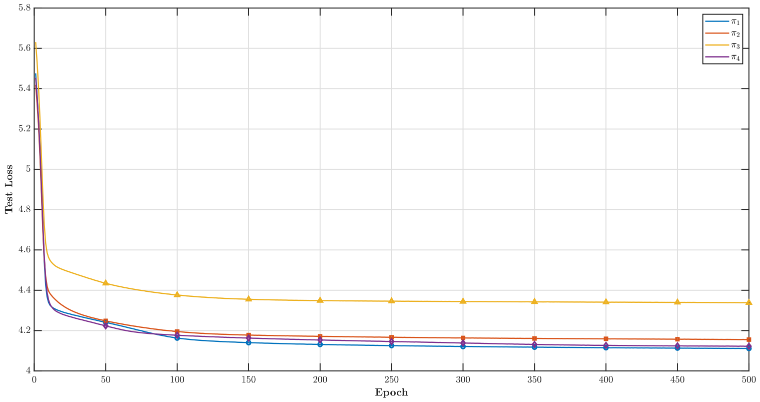

Fig. B. Comparison. Test errors of autoregressive models under different variable permutations.
