# Supplementary material for: Bayesian-calibrated global sensitivity analysis for mathematical models using generative AI
Source: PLoS Comput Biol. 2026 Mar 16;22(3):e1013312. doi: 10.1371/journal.pcbi.1013312 (PMC13004599; doi:10.1371/journal.pcbi.1013312)
Supplement: S5 Appendix — Supplementary results presenting detailed parameter definitions and Bayesian calibration results, as well as effective sample size diagnostics for posterior samples used to train the diffusion model. (PDF) [file pcbi.1013312.s005.pdf]

S5 Appendix. Cellular Dynamic Model. In this supplement, we present a parameter table that includes both the calibrated and fixed parameters, along with their biological interpretations. Based on the calibration results, we provide the posterior predictions for the total T cell population of a single patient in Fig A.

Table A. Descriptions and calibration results of CAR-T model parameters.

Parameter	Description	MLE	95% Credible Interval
α	Inhibition of CAR-T cells	5.50×10^{-7}	Fixed
r	Tumor cell growth rate	0.176	Fixed
b	Inverse tumor carrying capacity	5.00×10^{-13}	Fixed
v	Cytotoxic effect saturation constant	0.305	Fixed
θ	Memory to effector conversion	6.00×10^{-6}	Fixed
a	Half-saturation constant	1000	Fixed
β	Death rate of infused cells	0.0803	(0.0103, 2.78)
η	Engraftment rate of injected cells	0.288	(0.0708, 0.495)
$r_{\min}$	Min. expansion rate	4.88×10^{-3}	$(1.51 \times 10^{-3}, 9.57 \times 10^{-3})$
p_1	Initial expansion rate	2.51	(1.30, 2.92)
p_2	Expansion duration regulation rate	2.71×10^{-9}	$(1.57 \times 10^{-14}, 7.63 \times 10^{-8})$
p_3	Expansion decay regulation	10.9	(5.78, 35.8)
A	Half-saturation constant	30.5	(21.5, 98.4)
ζ	Death rate of effector cells	0.114	(0.0617, 0.311)
ϵ	Conversion to memory cells	0.930	(0.506, 0.991)
λ	Exhaustion rate of effector cells	0.899	(0.483, 0.980)
μ	Death rate of memory cells	0.237	(0.104, 0.677)
δ	Death rate of exhausted cells	0.112	(0.0839, 0.694)
γ	Cytotoxic rate of functional cells	1.97	(1.78, 2.38)
ϕ	Data precision ($1/\phi$ is variance)	5.15×10^{-16}	$(2.41 \times 10^{-16}, 8.67 \times 10^{-16})$

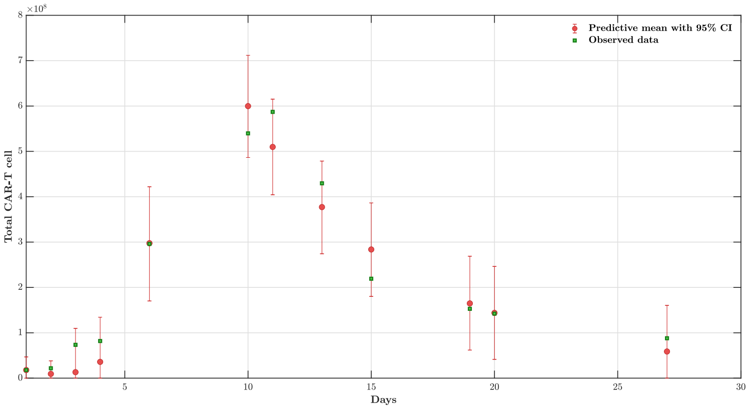

Fig. A. Result. Posterior predictive solutions for total CAR-T cell number

Using the posterior samples, we train diffusion models and subsequently implement Algorithm 2 to estimate the sensitivity indices. We present both the loss curves and the convergence diagnostics for the randomized Shapley estimators using RePaint-based conditional generation. The results are summarized in Fig B. For moderately high-dimensional inputs with complex parameter dependencies specified by the posterior distribution, it is important to determine a reasonable sample size that is sufficient to accurately capture the true posterior density. Fig C presents the learned posterior

density projected onto the β–γ plane using different sizes of samples drawn from the thinned posterior sample set. The results indicate that when the training sample size reaches approximately 15,000, the diffusion model is able to produce a reasonably accurate representation of the original posterior distribution.

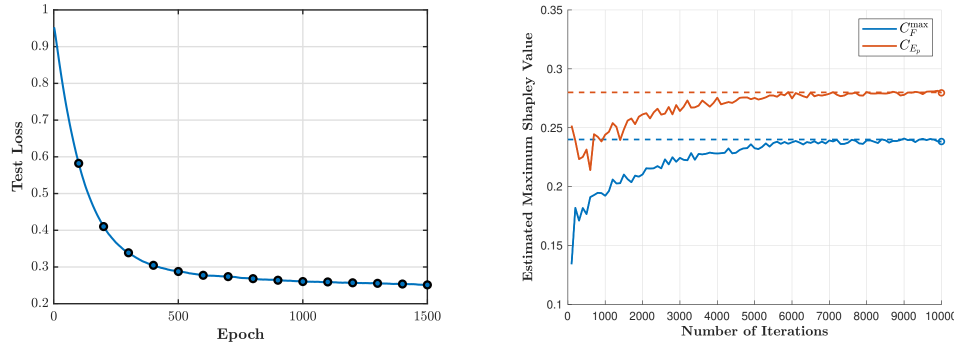

A. Test error **B.** Convergence test

Fig. B. Results. Diffusion model training results and convergence analysis of sensitivity indices using Algorithm 2.

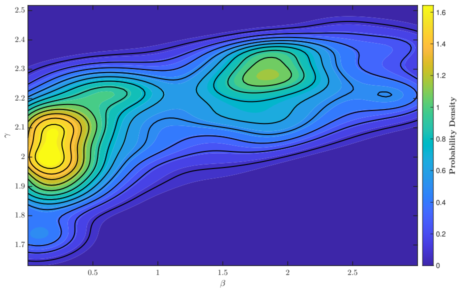

A. True posterior density

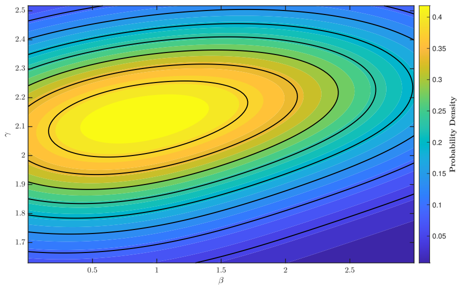

B. $N_1 = 5{,}000$

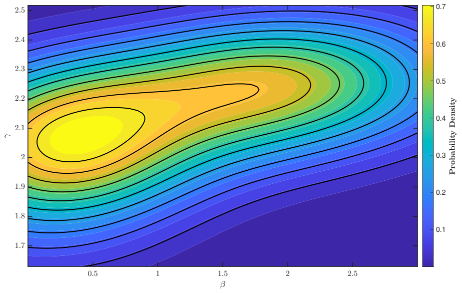

C. $N_1 = 10{,}000$

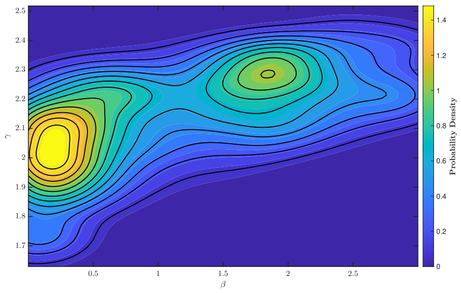

D. $N_1 = 15{,}000$

Fig. C. Comparison. Diffusion model training accuracy in approximating the true posterior density for different training sample sizes N_1.
