# Supplementary material for: Bayesian-calibrated global sensitivity analysis for mathematical models using generative AI
Source: PLoS Comput Biol. 2026 Mar 16;22(3):e1013312. doi: 10.1371/journal.pcbi.1013312 (PMC13004599; doi:10.1371/journal.pcbi.1013312)
Supplement: Attachment — Submitted filename: ploscomp-review-4112025.pdf [file pcbi.1013312.s006.pdf]

Re: Data-informed global sensitivity analysis for mathematical models using generative AI

In this manuscript, the author presents an innovative generative modelling framework for global sensitivity analysis (GSA) in complex, data-informed mathematical models. The modelling addresses the limitations in classical variance-based GSA by developing a Bayesian-compatible, generative AI-based approach that captures parameter dependencies directly from posterior samples. The manuscript is generally well-written with, with only a few minor grammar errors. I believe additional work is needed to strengthen the body of research presented in this study:

Comments:

1. The author did not provide a quantitative benchmark comparing computational performance—such as runtime, sample efficiency, or accuracy—of the new approach against baseline methods. A benchmark table demonstrating scalability or accuracy improvements over standard methods would be valuable. Note that the results in Table 1 are not sufficient, as Sobol indices do not appear to differ significantly from those of the analytical method (if by "analytical method", does the author meant the classical approach?).
2. The RePaint algorithm used in the diffusion model's conditional sampling is not mathematically guaranteed to produce exact conditional distributions. Although the author acknowledges this limitation, quantifying or visualising the approximation bias introduced by RePaint would strengthen the study.
3. The autoregressive component assumes Gaussian conditional distributions. Please include a test case or discussion addressing the model's robustness—or its limitations—when posterior marginals are non-Gaussian.
4. It is surprising that the data pipeline and AI model architecture used in the autoregressive model section were not described, and that software packages employed for implementation were not cited. Please include this information.
5. In the Abstract, change "Our methods addresses" to "Our method addresses."
6. Clarify the distinction between "data-informed" and "Bayesian-calibrated" models.
7. Add comparison plots showing posterior vs generative model distributions.
8. Please decide if Figure's caption should be above or below actual figures. (I recommend placing captions below the figures for consistency with journal conventions.)
9. The author has not convincingly demonstrated whether this approach performs better than existing methods. Is there an example where traditional variance-based GSA fails due to the assumption of independent inputs? Testing the new method against such examples would help establish the reliability and practical advantage of the proposed framework.
