# Supplementary material for: Bayesian-calibrated global sensitivity analysis for mathematical models using generative AI
Source: PLoS Comput Biol. 2026 Mar 16;22(3):e1013312. doi: 10.1371/journal.pcbi.1013312 (PMC13004599; doi:10.1371/journal.pcbi.1013312)
Supplement: Attachment — Submitted filename: Response to reviewers.pdf [file pcbi.1013312.s007.pdf]

Response to Reviewers

Manuscript ID: PCOMPBIOL-D-25-01368

Title: *Data-informed global sensitivity analysis for mathematical models using generative AI*

Submitted to: *PLOS Computational Biology*

Dear Editors and Reviewers,

I want to thank the Academic Editor, Section Editor, and the reviewers for their careful evaluation of the manuscript and for their constructive and insightful comments. I am encouraged by the positive assessment of the manuscript's overall quality and organization. Below, I provide a detailed, point-by-point response to all reviewer comments and describe how the manuscript has been revised accordingly. All changes are highlighted in the revised manuscript using track changes. In addition, to comply with the journal's formatting requirements, a clean final version without highlighted changes is also provided in the journal's LaTeX format.

Response to Reviewer #1

Comment 1: *The novelty of the proposed method should be more clearly articulated. Beyond the direct integration of generative models into the formulations of Rosenblatt transformations and Shapley effects, are there any methodological innovations or theoretical insights that distinguish this work?*

Response: I appreciate this important comment. I would like to clarify that the main novelty of this work lies in a methodological innovation that incorporates generative AI into global sensitivity analysis. In the revised manuscript, I explicitly clarify that the main contribution is to reframe sensitivity analysis as a post Bayesian calibration task for biological modeling problems. Within this framework, generative models serve as effective surrogates for the calibrated posterior distribution, enabling accurate, data-driven estimation of input sensitivities. The key advantage of the proposed approach is that the generative models avoid explicit copula construction and restrictive parametric assumptions, thereby allowing sensitivity analysis to be conducted in a data-relevant and realistic manner under complex dependency structures. I have revised the Abstract, Author Summery and Introduction sections to clearly articulate these contributions and to distinguish our approach from existing copula based and surrogate based GSA methods.

Comment 2: *The generative models are trained using MCMC samples, which are inherently dependent. The authors are recommended to discuss how this dependency affects model training and performance, such as in terms of convergence behavior, sampling efficiency, and the reliability of the learned distributions.*

Response: I thank the reviewer for highlighting this important practical issue. I have added a discussion on the impact of MCMC dependence on the training of generative models with related references. I note that although MCMC samples are inherently correlated, the generative models used in this study are generally robust provided that the MCMC chains are moderately thinned to reduce autocorrelation. Evidence from the numerical experiments further indicates stable training behavior and accurate reproduction of both joint and conditional distributions. This discussion has been added to the Methods section and is further supported by new results presented in the supplementary materials.

Comment 3: *A key concern is the limited quantitative validation of the proposed method. Currently, only a low-dimensional linear model (three variables) is used for quantitative assessment. Given that high-dimensional settings are a primary motivation for this work, it is strongly recommended to include quantitative validations for higher-dimensional problems to substantiate the applicability and robustness.*

Response: I agree with the reviewer that stronger quantitative validation in higher-dimensional settings is important. In response, I have added an additional numerical experiment in the Case Study section involving a 200-dimensional Sobol benchmark function to quantitatively evaluate both the accuracy and the computational efficiency of the proposed method. The results show that the proposed approach achieves sensitivity estimates with accuracy comparable to well-established existing methods as dimensionality increases, without introducing excessive computational cost. These new results are presented in the Case Study section and also in supplementary materials.

Comment 4: *Another major concern is about the sample size required to make the proposed method effective. In the demonstrative cases, the samples used to train the generative models are actually pretty large. Specifically, 50,000 samples, 100,000 samples, and 1,000,000 samples for the first, second, and third cases, respectively. However, the dimension of the involved parameters is not too high (no more than 20). It is important to examine how the performance of the proposed method scales with respect to sample size. A sensitivity analysis on the number of training samples would help clarify the practical feasibility of this approach.*

Response: I appreciate this insightful suggestion. My original intent was to use sufficiently large training sample sizes and network capacities to ensure accurate estimation of the sensitivity indices. However, I agree that understanding the minimal sample size required for reliable performance is an important practical consideration.

To address this point, I have added additional numerical experiments examining the effects of network size and training sample size on performance. These results are provided for the new Ishigami function example with low-dimensional but complex input distributions, as well as for the cellular dynamical model presented later in the Case Study section. I find that a relatively small network architecture (2×32) is sufficient across all numerical examples, and that good performance is already achieved with approximately 10,000 training posterior samples.

To ensure that the sensitivity estimates remain accurate when using smaller training sets and to quantify the uncertainty associated with limited sample sizes, I further introduce a bootstrapping procedure for each numerical example. The resulting bootstrap mean are shown to be very close to those obtained using large training samples, providing a more informative and robust presentation of the results. These findings are now included in the Case Study section of the revised manuscript and in the supplementary materials.

Response to Reviewer #2

The author did not provide a quantitative benchmark comparing computational performance—such as runtime, sample efficiency, or accuracy—of the new approach against baseline methods. A benchmark table demonstrating scalability or accuracy improvements over standard methods would be valuable. Note that the results in Table 1 are not sufficient, as Sobol indices do not appear to differ significantly from those of the analytical method (if by "analytical method", does the author meant the classical approach?)

Response: I thank the reviewer for this valuable suggestion. In the revised manuscript, I have added two additional quantitative benchmarks that compare both the accuracy and efficiency of the

proposed method with three widely used GSA methods. The benchmark examples span a range of input dimensions and correlation structures. The resulting tables explicitly demonstrate scalability and highlight the computational trade-offs involved in achieving improved accuracy through the additional cost of learning correlation-aware generative surrogates.

I also clarify that the term “analytical method” refers to theoretical reference values, namely sensitivity indices that can be obtained either in closed form or under known input distributions and therefore serve as ground truth for validation. These additional results have been incorporated into the Case Study section as well as the supplementary materials.

The RePaint algorithm used in the diffusion model’s conditional sampling is not mathematically guaranteed to produce exact conditional distributions. Although the author acknowledges this limitation, quantifying or visualising the approximation bias introduced by RePaint would strengthen the study.

Response: I appreciate this important observation. In response, I have added both quantitative and visual assessments of the approximation bias introduced by the RePaint conditional sampling procedure, using the new benchmark examples based on the Ishigami function and the Sobol g-function. Specifically, I compare conditional distributions generated by RePaint with reference conditional distributions obtained from ground-truth input models, using density plots and heatmap-based error metrics. The results indicate that, while RePaint provides an approximate conditional sampler, it yields sufficiently accurate samples for sensitivity estimation in the considered settings. This analysis is now included in the supplementary materials.

The autoregressive component assumes Gaussian conditional distributions. Please include a test case or discussion addressing the model’s robustness—or its limitations—when posterior marginals are non-Gaussian.

Response: I thank the reviewer for raising this point. I would like to clarify that the proposed Gaussian-based autoregressive model is combined with a standard normal cumulative distribution function to map the modeled distributions onto the unit hypercube, thereby approximating the Rosenblatt transformation. In this context, the term “non-Gaussian” primarily refers to asymmetric or multimodal distributions.

That said, the reviewer is absolutely correct in identifying the limitations of this approach. In the revised manuscript, I have added comparison plots for highly asymmetric and multimodal input distributions, which clearly demonstrate that the autoregressive model may fail to accurately capture such complex marginals and conditional distributions. These limitations are now explicitly discussed, and the corresponding results are provided in the supplementary materials.

It is surprising that the data pipeline and AI model architecture used in the autoregressive model section were not described, and that software packages employed for implementation were not cited. Please include this information

Response: I agree with the reviewer that this information is essential. In the revised manuscript, I now provide a detailed description of the data preprocessing pipeline, the autoregressive model architecture, and the associated training procedures. I also explicitly cite the software packages used for implementation. These additions are primarily included in the Autoregressive Model section.

In the Abstract, change “Our methods addresses” to “Our method addresses.”

Response: Thank you for pointing out this typographical error. I have corrected this in the revised Abstract.

Clarify the distinction between “data-informed” and “Bayesian-calibrated” models

Response: I appreciate this request for clarification. Upon revision, I recognized that “Bayesian-calibrated” more accurately describes the proposed framework. Accordingly, I have replaced the term “data-informed” with “Bayesian-calibrated” in the title, Author Summary, and Introduction to ensure consistent and precise terminology throughout the manuscript.

Add comparison plots showing posterior vs generative model distributions.

Response: I thank the reviewer for this suggestion. In response, I have added comparison plots that directly contrast posterior samples with samples generated by the trained generative models, including both marginal and pairwise distributions. These plots are now provided in the supplementary materials.

Please decide if Figure's caption should be above or below actual figures. (I recommend placing captions below the figures for consistency with journal conventions.)

Response: Thank you for this recommendation. All figures have been revised so that captions are consistently placed below the figures, and all table captions are positioned above the corresponding tables, in accordance with the journal's conventions.

The author has not convincingly demonstrated whether this approach performs better than existing methods. Is there an example where traditional variance-based GSA fails due to the assumption of independent inputs? Testing the new method against such examples would help establish the reliability and practical advantage of the proposed framework.

Response: I appreciate this important comment. In the revised manuscript, I have included additional experiments that explicitly demonstrate scenarios in which traditional variance-based GSA methods fail due to violations of the input independence assumption.

Specifically, I identify two major failure modes. First, traditional methods relying on restrictive parametric assumptions are unable to capture complex input dependencies, leading to significantly biased sensitivity estimates. This issue is demonstrated in the revised Ishigami example with complex correlated inputs. Second, traditional approaches fail to represent data-driven posterior correlations arising from Bayesian calibration, which can result in conditional variances that exceed posterior predictive variances, thereby invalidating Sobol indices. This phenomenon is illustrated in the updated SIR model example (new Figure 2), where sample swapping destroys the calibrated dependency structure and leads to unrealistically large output variability. Together, these examples explicitly demonstrate the practical advantage and reliability of the proposed framework in settings where standard methods are not applicable.

Once again, I sincerely thank the editors and reviewers for their constructive feedback. I believe that the revisions have substantially strengthened the manuscript and clarified both the novelty and practical applicability of the proposed approach. I hope that the revised version is now suitable for publication in *PLOS Computational Biology.*

Sincerely,

Xuyuan Wang
