# Supplementary material for: Bayesian-calibrated global sensitivity analysis for mathematical models using generative AI
Source: PLoS Comput Biol. 2026 Mar 16;22(3):e1013312. doi: 10.1371/journal.pcbi.1013312 (PMC13004599; doi:10.1371/journal.pcbi.1013312)
Supplement: Attachment — Submitted filename: Response_to_Reviewers_auresp_2.pdf [file pcbi.1013312.s008.pdf]

Response to Reviewers

Manuscript ID: PCOMPBIOL-D-25-01368

Title: *Bayesian-calibrated global sensitivity analysis for mathematical models using generative AI*

Submitted to: *PLOS Computational Biology*

Dear Editors and Reviewers,

I would like to thank you for your careful evaluation of the manuscript. I have addressed all comments and suggestions as detailed below.

Response to Reviewer #2

Figure 2 in the manuscript. The fonts and ticks are too small.

Response: I thank the reviewer for this helpful suggestion. Figure 2 has been revised by increasing the font sizes and tick label sizes to improve readability. I would be happy to make further adjustments if additional improvements are recommended.

Sincerely,

Xuyuan Wang
